# Chemical Composition and Antimicrobial Activities of Essential Oils of Some Coniferous Plants Cultivated in Egypt

**Published:** 2017

**Authors:** Taghreed A. Ibrahim, Atef A. El-Hela, Hala M. El-Hefnawy, Areej M. Al-Taweel, Shagufta Perveen

**Affiliations:** a *Department of Pharmacognosy, College of Pharmacy, King Saud University, P.O. Box 2457, Riyadh 11451, Saudi Arabia.*; b *Departmaent of Pharmacognosy, Faculty of Pharmacy, Cairo University, Cairo, Egypt.*; c *Department of Pharmacognosy, Faculty of Pharmacy, Al – Azhar University, Nasr City, Cairo, Egypt.*

**Keywords:** *Calocedrus*, Cupressaceae, *Cupressus*, monoterpene, sesquiterpene, *Tetraclinis*

## Abstract

Family Cupressaceae is the largest coniferous plant family. Essential oils of many species belonging to family Cupressaceae are known to have several biological activities specially antimicrobial activity. The essential oils from aerial parts of *Calocedrus decurrens *Torr.,* Cupressus sempervirens stricta *L. and* Tetraclinis articulata* (Vahl) Mast. were prepared by hydrodistillation. The chemical composition of the essential oils has been elucidated by gas chromatography-mass spectroscopy analysis. The prepared essential oils were examined against selected species of Gram-positive, Gram-negative bacteria and *Candida* species. Broth dilution methods were used to detect minimum inhibitory concentration (MIC), minimum bactericidal concentration (MBC) and minimum fungicidal concentration (MFC). Sixteen compounds were identified in the essential oils of both *Calocedrus decurrens *and *Cupressus sempervirens *L. and fifteen compounds were identified in the essential oil of *Tetraclinis articulata*. δ-3-Carene (43.10%), (+)-Cedrol (74.03%) and Camphor (21.23%) were the major constituents in the essential oils of *Calocedrus decurrens*, *Cupressus sempervirens *L. and *Tetraclinis articulata*, respectively. The essential oils showed strong antimicrobial activities against the selected microorganisms in concentration range 0.02 3- 3.03 µL/mL. This study could contribute to the chemotaxonomic characterization of family Cupressaceae. In addition, it proved that the essential oils under investigation possess potential antimicrobial properties.

## Introduction

The use of essential oils to control many diseases and their effective usage as antimicrobial agents (1-5) in addition to their use as functional ingredients in foods, drinks, toiletries and cosmetics is gaining momentum, both for the growing interest of consumers in ingredients from natural sources and also because of increasing concern about potentially harmful synthetic additives (6). Essential oils are complex mixture of natural compounds, mostly of plant origin, extremely volatile and with an intense odour. Even if they represent only a small fraction of the plant from which they are derived, they give the whole plant the characteristic of aromatic smell for which these plants are employed by drug, food and perfume industries (7). The species that show the largest content of essential oils belong to many families as Asteraceae, Lamiaceae, Apiaceae, Rutaceae, Lauraceae, Myrtaceae, Magnoliaceae, Pinaceae and Cupressaceae (8-10).

Family Cupressaceae is a common ornamental plants, cultivated around the world, and particularly in South America, Mediterranean basin and North Africa, It is the most widely distributed of all gymnosperm families, occurring in diverse habitats on all continents. Cupressaceae is also the most important conifer family in modern horticulture, members of this family are important for their timber, resin, fruit and as ornamentals (11-13).

Several species belonging to family Cupressaceae have been used in folk medicine as astringent, antiseptic, pulmonary antiseptic, antispasmodic, lymphatic tonic, antibacterial, antihemorrhagic, capillary protector, antipyretic and pelvic decongestant (14). Many biologically active compounds have been reported from Cupressaceae species such as essential oils, diterpenes, flavonoids, and sterols (14, 15). 

Genus *Cupressus* comprising about 20 species;* Cupressus sempervirens *L. commonly known as Cypress and known in Arabic as Sarȗ, Sarw, Serwal, Sharbin and Shagaret el Hayat (16), is native to eastern North America (17) and grown in Egypt as ornamental tree. The essential oil of the leaves of the plant cultivated in Alexandria, Egypt was studied; the main oil constituents were cedrol (21.29%), δ-3-carene (17.85%) and α-pinene (6.90%) and proved to have antimicrobial activities (18). Essential oil of the aerial parts of* Cupressus sempervirens *cultivated in Safx, Tunisia was studied, 24 compounds were identified, where α-pinene (37.14%), δ-3-careen (19.67%) and R (+)-limonene (5.43%) were the main oil constituents, the oil exhibited a significant antimicrobial activities (19).

*Calocedrus decurrens* (Incense Ceder) is a tree native to Oregon and California, known for its aromatic wood and manufacturing of pencils (20)]. Heartwood essential oil of *C. decurrens* was studied, it was found that *p*-cymene and *p*-menthane derivatives were the main components (20). Von Rudloff (21) reported that leaf oil of *C. decurrens* from the pacific northwest, USA, was dominated by limonene (31.31%), δ-3-carene (21%) and α-pinene (9.2%) while Robert & Sanko (22) proved that the oils of *C. decurrens* from two populations in Oregon and one disjunct population in southern California were high in δ-3 carene (15.20%- 20.00%), limonene (18.21-23.62%), α-pinene (8.7-15.8%), terpinolene (5.72- 8.01%), α-fenchyl acetate (3,5- 9.71%) and cedrol (0.8- 1.2%) (21,22).

*Tetraclinis articulata* (Cartagena Cypress, known in Arabic as Ahrar, Berbouch and Megloub (23) is an endemic species of North Africa, Malt and Spain (24). Various parts of this tree are used in folk medicine for its multiple therapeutic effects, it is mainly used against childhood respiratory and intestinal infections (24)], gastric pains, diabetes, hypertension and used as antidiarrheal, antipyretic, diuretic, antirheumatic and oral hypoglycemic (24-26). Twenty-seven compounds were detected in Algerian *T. articulata* leaves, bornyl acetate (52.11%), caryophyllene (7.51%), germacrene D (5.61%) and caryophyllene oxide (5.01%) were the major components, the oil exhibited antifungal (27), antibacterial (28), antioxidant and anti-inflammatory activities (29).

Fifty-Six compounds were detected in Tunisian *T. articulata* plant, where α-pinene (31.32%), linalool acetate (18.18%), alloaromadendrene (7.55%) and γ-caryophyllene (4.16%) were the major constituents in the oil prepared by hydrodistillation method, the oil exhibited antioxidant activities (30, 31).

 The main objective of this study was to identify the chemical composition of the essential oils from the aerial parts of *C. decurrens*, *C. sempervirens* and *T. articulata* cultivated in Egypt, and to determine their antimicrobial activities, in an attempt to contribute to the use of these essential oils as alternative products.

## Experimental

*Plant materials*

The aerial parts of *Calocedrus decurrens* Torr. (California Incense-Cedar), *Cupressus sempervirens *L. (Mediterranean or Italian Cypress) and *Tetraclinis articulata* (Vahl) Mast. (Cartagena Cypress) were collected from El-Orman Garden, Giza, Egypt on April 2012, at the flowering period. The identity of the plants was kindly verified by Terase Labib, General Manager and Specialist of Plant Taxonomy in El-Orman Botanical Garden, Giza, Egypt. Voucher specimens were deposited in the Department of Pharmacognosy, Faculty of Pharmacy, Al -Azhar University, Cairo, Egypt. 

*Preparation of the essential oil*

The essential oils were prepared from the fresh aerial parts of *C. decurrens, C. sempervirens *and* T. articulata *(500 g) by hydro distillation using Clevenger-type apparatus. The oils were dried over anhydrous sodium sulphate and stored in sealed glass vials at 4-6 ^o^C prior to analysis. Percentage yields were determined according to Egyptian Pharmacopoeia 1984 (32). Percentage yields and physical properties of the essential oils are illustrated in Table 1. 

*GC/ MS analysis of the essential oil*

The prepared essential oils were subjected to GC/MS analysis using Shimadzu GC//MS – QP 5050 A, fitted with a DB-1 fused silica capillary column (30 m, 0.53 mm ID, 1.51 µM film thickness). Software Class 5000. searched library: Wiley 229 LIB. Carrier gas: Helium (flow rate 1 mL/min.). Ionization mode: EL (70 ev). Temperature program: 40 ^0^C (static for 2 min) then gradually increasing at a rate of 2 ^0^C/min up to 250 ^0^C (static for 7.50 min). Detector temperature 250 ^0^C. Injector temperature 250 ^0^C.

*Identification of the essential oil*

Compounds were identified by comparison of their retention indices (RI), obtained on a non-polar DB-1 column relative to C5- C24 *n*-alkanes, with those provided in the literature, in addition to Library searched data base Willey 229LIB and by comparing mass fragmentation patterns with those of the available references and with published data (33-35). The percentage composition of the essential oils was determined by computerized peak area measurements. Results were calculated as mean values after two injections for each essential oil. Results are presented in Tables 2,3.

*Test organisms*

Pure strains of bacteria (*Staphylococcus aureus* ATCC 13709, *Staphylococcus epidermidis* ATCC 35984, *Streptococcus pyogenes *ATCC 19615*,* *Eschrechia coli* ATCC 9637,* Klebsiella pneumoniae* ATCC 1705,* Proteus vulgaris *ATCC 8427,* Pseudomonas aeruginosa* ATCC 27853 and* Shigella boydii* ATCC 9905) as well as pure strains of* Candida albicans* ATCC 10231, *Candida glabrata* ATCC 90030, *Candida krusei* ATCC 14243 and *Candida parapsilosis *ATCC 22019 were used. All micro-organisms were kindly supplied from the Microbiology Department, Faculty of Pharmacy, Al-Azhar University, Nasr City, Cairo, Egypt.

*Testing for antimicrobial activities*

Minimum Inhibitory Concentrations (MICs) were determined using the broth microdilution method (36, 37). Determination of antimicrobial activities against yeast was achieved by microdilution method using serial dilutions of the essential oils (0.008–64.00 *μ*L/mL), which were prepared in 96-well microtiter plates by microdilution method using RPMI-1640 media (Sigma, St. Louis, MO, USA) buffered with MOPS (Sigma). The antibacterial activities were determined by serial dilutions of the essential oils (0.03–128.00 *μ*L/mL, DMSO) in Mueller–Hinton broth (Merck, Darmstadt, Germany). Test yeasts or bacteria strains were suspended in media and the cell densities were adjusted to 0.5 McFarland standards at 530 nm wavelength using a spectrophotometeric method. Inoculums (0.1 mL) were added to the microtiter plates, which were incubated in a humid atmosphere at 30 °C for 24–48 h (yeast) or at 37 °C for 24 h (bacteria). In addition, positive (medium with inoculums but without essential oil) and negative (Uninoculated medium, 200 μL) growth controls were prepared. The growth in each well was compared with the growth in the control well. MICs were visually determined and defined as the lowest concentration of the essential oil produced ≥ 50% growth inhibition for fungi and ≥95% growth reduction for bacteria compared with the growth in the control well. Each experiment was performed in triplicate. Gentamycin (Sigma-Aldrich, Steinheim, Germany) and nystatin (Merck, Darmstadt, Germany) in concentration range (0.001- 64.00 µg/mL, sterile distilled water) were used as standard antibacterial and antifungal drugs, respectively.

In addition, media from wells with fungi showing no visible growth were further cultured on Sabouraud dextrose agar (Merck, Darmstadt, Germany) and from wells with bacteria showing no visible growth on Mueller-Hinton agar (Merck, Darmstadt, Germany) to determine the minimum fungicidal concentrations (MFC) and minimum bactericidal concentrations (MBC).

*Statistical Analysis*

Results were expressed as the mean ± standard deviation; statistical analysis of experimental results were based on the analysis of variance method. Differences were considered statistically significant at the level of P < 0.001.

## Results and Discussion

A noticeable variation was observed in the percentage yield of hydrodistilled essential oil prepared from the aerial parts of calocedrus incense cedar,* C. sempervirens*, and *T. articulata,* cultivated in Egypt yielding (1.41%, 0.32% and 1.71%), respectively. Results of GC/MS analysis of essential oils of plants under investigation showed qualitative and quantitative variations. Sixteen compounds were determined in essential oils of* C.* *decurrens *and* C. sempervirens*, while fifteen compounds were identified in essential oil of* T. articulata.*

**Table 1:** Yield and physical properties of essential oils prepared from *C.* *decurrens*, *C. sempervirens* and *T. articulata* fresh aerial parts

**Plant **	***C. decurrens ***	***C. semervirens ***	***T .articulata ***
Essential oils (g % of fresh weight)	1.42	0.30	1.71
Refractive index at 25 ^0^C	1.343	1.463	1.399
Specific gravity	0.8884	0. 8879	0.8921

**Table 2:** Chemical composition of essential oils of *C.* *decurrens*, *C. sempervirens* and *T. articulata* aerial parts

**No.**	**R**_t _ **(min.)**	**KI** *	**Compound**	**Percentage (%)**		
				***C.*** ***decurrens***	***C. sempervirens***	***T. articulat*** ***a***
1	11.5	834	Isovaleric acid	-	0.73	-
2	12.3	934	α-pinene	2.59	4.60	5.92
3	12.8	1000	Decyne	-	0.57	-
4	13.5	1011	δ-3-Carene	43.10	3.80	-
5	13.7	1026	*p*-Cymene	2.56	-	1.65
6	14.3	1047	(R)-(+)-Limonene	0.74	-	3.00
7	14.9	1062	γ-terpinene	2.67	-	-
8	15.6	1075	Fenchone	-	-	9.48
9	16.2	1088	Terpinolene	3.74	0.31	-
10	17.2	1098	Linalool	3.91	-	-
11	17.8	1102	(-)-α-Thujone	1.84	0.30	-
12	18.7	1120	α-Fenchol	13.07	-	-
13	19.2	1123	(+)-Fenchol	-	-	13.85
14	20.6	1143	Camphor	-	-	21.23
15	21.5	1165	(-)-Borneol	-	2.33	-
16	23. 7	1185	(+)-α-Terpineol	-	0.50	3.12
17	24.4	1221	α-Fenchyl acetate	14.16	-	4.83
18	25.6	1262	Chrysanthenyl acetate	-	-	3.30
19	26.6	1285	Bornyl acetate	-	-	15.03
20	26.8	1287	Isobornyl acetate	-	-	8.39
21	27.5	1290	Thymol	0.79	4.25	-
22	28.2	1352	α-Terpenyl acetate	-		3.47
23	28.4	1370	(+)-Curcuphenol	-	0.45	-
24	29.8	1418	β-Caryophyllene	1.72	-	3.51
25	30.2	1461	(−)-allo-aromadendrene	0.65	-	1.75
26	31.8	1495	Zingiberene	-	0.93	-
27	35.8	1576	Spathulenol	-	0.92	1.47
28	36.9	1581	(-)-Caryophyllene oxide	-	3.31	-
29	37.5	1596	(+)-Cedrol	4.51	74.03	-
30	38.9	1653	α-Cadinol	2.25	2.19	-
31	43.01	1984	Palmitic acid	1.70	-	-
32	47.75	2200	Stearic acid	-	0.78	-

* Kovats Index on DB-1 column in reference to *n*-alkanes

**Table 3:** Classification of essential oils constituents of *C. decurrens*, *C. sempervirens* and *T. articulata* aerial parts and their percentages

**Class**	**Percentage of constituents (%)**		
	***C. decurrens***	***C. sempervirens***	***T. articulata***
1-Monoterpenes			
Oxygenated	33.77	7.38	82.70
Non Oxygenated	55.40	9.28	10.57
2-Sesquiterpenes			
Oxygenated	6.76	80.90	1.47
Non Oxygenated	2.37	0.93	5.26
3-Fatty acids	1.70	0.78	-
4-Misceleneous group	-	0.73	-

**Table 4:** Antibacterial activities (MIC and MBC) of *C.  decurrens*, *C. sempervirens* and *T. articulata* essential oils

**Microorganism**	**Mean (µL/mL) ± Standard Deviation**						**Gentamycin ** **Mean (µg/mL) ± Standard Deviation**	
	***C. *** *** decurrens***		***C.*** *** sempervirens***		***T. articulata***			
	MIC90	MBC	MIC90	MBC	MIC90	MBC	MIC90	MBC
**Gram-positive**								
*Staphylococcus aureus*	< 64	-	1.148 ± 0.067	3.031± 0.093	1.319 ± 0.074	3.031 ± 0.18	0.02 ± 0.004	1 ± 0.023
*Staphylococcus epidermidis*	0.442 ± 0.027	2.244 ± 0.092	0.047 ± 0.002	0.391± 0.073	0.023 ± 0.009	0.155 ± 0.062	0.02 ± 0.003	1 ± 0.046
*Streptococcus pyogenes*	< 64	-	0.84 ± 0.064	2.378± 0.13	< 64	-	0.02 ± 0.003	1 ± 0.028
**Gram-negative**								
*E. coli*	< 64	-	0.037 ± 0.006	0.155 ± 0.052	0.659 ± 0.063	1.515 ± 0.052	0.04 ± 0.002	2 ± 0.027
*Klebsiella pneumonia*	0.500 ± 0.031	1.023± 0.11	1.319 ± 0.056	1.64 ± 0.080	2.828 ± 0.076	4.49 ± 0.24	0.02 ± 0.003	1 ± 0.027
*Proteus vulgaris*	1.414 ± 0.072	5.04 ± 0.094	1.414 ± 0.051	2.828 ± 0.160	3.031 ± 0.058	4.59 ± 0.095	0.02 ± 0.003	1 ± 0.050
*Pseudomonas aeruginosa*	1.515± 0.042	2.297 ± 0.056	0.42 ± 0.035	1.00 ± 0.062	2.378 ± 0.052	6.72 ± 0.27	0.037 ± 0.004	2 ± 0.032
*Shigella boydii*‎	0.435± 0.054	1.148 ± 0.072	0.84 ± 0.035	2.378 ± 0.120	0.757 ± 0.036	1.00 ± 0.064	0.02 ± 0.001	1 ± 0.032

-Not done

**Table 5:** Antifungal activities (MIC and MFC) of *C.  decurrens*, *C. sempervirens* and *T. articulata* essential oils

**Microorganism**	**Mean (µL/mL) ± Standard Deviation**						**Nystatin ** **Mean (µg/mL) ± Standard Deviation**	
	***C. *** *** decurrens***		***C.*** *** sempervirens***		***T. articulata***			
	**MIC90**	**MFC**	**MIC90**	**MFC**	**MIC90**	**MFC**	**MIC90**	**MFC**
*Candida albicans*	< 64	-	0.42 ± 0.027	1.319 ± 0.066	0.659 ± 0.053	1.515 ± 0.085	0.84 ± 0.062	1 ± 0.062
*Candida glabrata*	< 64	-	< 64	-	< 64	-	0.84 ± 0.032	1 ± 0.045
*Candida krusei*	< 64	-	< 64	-	< 64	-	1.148 ± 0.065	2 ± 0.034
*Candida parapsilosis*	0.824 ± 0.052	2.828 ± 0.182	0.757 ± 0.067	1.64 ± 0.058	0.659 ± 0.042	1.148 ± 0.087	0.84 ± 0.076	1 ± 0.041

-Not done

Table 2. showed that δ-3-carene (43.10%), (+)-cedrol (74.03%) and camphor (21.23%) were the major constituents of the essential oils of *C. decurrens*, *C. sempervirens* and *T. articulata*, respectively. In addition alpha- fenchyl acetate (14.16%) and α- fenchol (13.07%) were predominant in essential oil of *C*. *decurrens*; α-pinene (4.60%) and δ-3-carene (3.80%) were dominated in essential oil of *C. sempervirens* and *T. articulata* essential oil showed the presence of bornyl acetate and and (+)-fenchol in a percentage of 15.03% and 13.85%, respectively. 

Tables 2,3. showed that the highest percentage of oxygenated monoterpenes was observed in the essential oil of *T. articulata* (82.70%) followed by *C. decurrens *(33.77%)* and C. sempervirens* (7.38%). Camphor (21.23%) was the major oxygenated monoterpene in essential oil of *T. articulata* followed by bornyl acetate (15.03%), while α-fenchyl acetate (14.16%) and α-fenchol (13.07%) were the major oxygenated monoterpenes in the essential oil of *C. decurrens*. Whereas, thymol (4.25%) and borneol (2.33%) were the major oxygenated monoterpenes detected in the essential oil of *C. sempervirens.*

Essential oil of* C. decurrens* showed the highest percentage of non-oxygenated monoterpenes (55.40%) followed by *T. articulata *(10.57%) and *C. sempervirens* (9.28%). δ-3-carene (43.10%) represented the major non-oxygenated monoterpene in the essential oil of *C. decurrens* followed by terpinolene (3.74%). α-pinene (5.92%) was the major non-oxygenated monoterpene in the essential oil of *T. articulata *followed by limonene (3.00%). While the main non-oxygenated monoterpene in the essential oil of *C. sempervirens* was α -pinene (4.60%) followed by δ-3-carene (3.80%). Essential oil of* C. sempervirens* showed the highest percentage of oxygenated sesquiterpenes (80.90%) followed by *C. decurrens* (6.76%) and *T. articulata *(1.47%). (+)-Cedrol (74.03%) was the main constituent of the oxygenated sesquiterpenes of the essential oil of *C. sempervirens* followed by caryophyllene oxide (3.31%), also (+)-cedrol (4.51%) constituted the main oxygenated sesquiterpenes of the essential oil of *C. decurrens* followed by α-cadinol (2.25%), while spathulenol (1.47%) was the only oxygenated sesquiterpene detected in the essential oil of *T. articulata*. Essential oil of* T. articulata *showed the highest percentage of non-oxygenated sesquiterpenes (5.26%) followed by *C. decurrens* (2.37%) and *C. sempervirens* (0.93%). Zingibrene (0.93%) was the only detected sesquiterpene hydrocarbon in the essential oil of *C. sempervirens* while, β-caryophyllene (1.72% and 3.51%) and allo-aromadanderene (0.65% and 1.75%) were the only detected non-oxygenated sesquiterpenes in the essential oils of *C. decurrens* and *T. articulata*, respectively.

The antibacterial activities of *C. decurren*s, *C. sempervirens* and *T. articulata *essential oils against the tested Gram-positive and Gram- negative baeteria are shown in Table 4. The essential oils under investigation inhibited the growth of *S. epidermidis* at concentrations 0.023- 0.442 µL/mL. Essential oil of *C. sempervirens* showed inhibition of the growth of *S. pyogenes* at concentration 0.84 µL/mL, while essential oils of *C. decurren*s and *T. articulata *at concentrations up to 64 µL/mL showed no inhibition of the growth of *S. pyogenes*. *E. coli* showed no susceptibility to essential oil of *C. decurren*s, while all the tested Gram-negative microorganisms showed growth inhibition by the effect of essential oils of *C. sempervirens* and *T. articulata *at concentrations range 0.037- 3.031 µL/mL. In addition, all the tested essential oils excreted bactericidal activities against all the susceptible Gram-positive and gram-negative microorganisms at concentration range 0.155- 6.72 µL/mL. *C. sempervirens* showed the highest antibacterial activities against most of the tested bacterial strains.

The antifungal activities of the essential oils of *C. decurren*s, *C. sempervirens* and *T. articulata *against tested yeast strains are shown in Table 5. Essential oil of *C. decurren*s showed no activities against all the tested yeast strains except *C. parapsilosis* which showed growth inhibition at concentration 0.824 µL/mL. *C. glabrata* and *C. krusei* showed no susceptibility to any of the studied essential oils, while essential oils of *C. sempervirens* and *T. articulata *inhibited the growth of *C. albicans* and *C. parapsilosis* at concentration range 0.42- 0.757 µL/mL. The tested essential oils showed MFC against the susceptible *Candida* species ranging from 1.148 µL/mL to 2.828 µL/mL. *C. sempervirens* essential oil showed the highest fungicidal activities followed by *T. articulata *and *C. decurren*s*. *

From this study it could be concluded that the essential oils under investigation possess antimicrobial activities. *C. sempervirens *essential oil has the most potential antimicrobial properties followed by *T. articulata* essential oil. The results of the study are inaccordance with the previous investigations of essential oil of *T. articulata*, which proved the presence of α-pinene, camphor, linalool acetate, caryophyllene, alloaromadendrene, bornyl acetate and limonene as the major constituents in several studies of essential oil of different organs of *T. articulata* in different countries (27-31, 38, 39). Previous investigations on essential oil of *C. decurren*s from USA and Taiwan proved the presence of α-pinene, δ-3-carene, terpinene, terpinolene, linalool, α-fenchyl acetate, β-caryophyllene and cedrol (21, 22)] which were detected in this study. While, a previous study on essential oil of leaves of *C. sempervirens* cultivated in Egypt proved that cedrol constituted the major constituent of the oil followed by δ-3-carene and α-pinene (18) whilst the essential oil of the leaves of the plant cultivated in Tunisia showed the presence of α-pinene as a major component followed by δ-3-carene and limonene (19). As the composition of the essential oils revealed intraspecific chemical variability among the same species growing in different localities and different environmental conditions, this study could contribute to the chemotaxonomic characterization of family Cupressaceae. 

From this study, it was concluded that the essential oils of plants of family Cupressaceae which were under investigation in this study showed low presence of non-oxygenated sesquiterpenes ranging from 0.93% to 5.26%. In addition to the occurrence of variable percentages of non-oxygenated monoterpenes (9.28%-55.4%), oxygenated monoterpenes (7.38%- 83.70%) and oxygenated sesquiterpenes (1.47%-80.90%). Meanwhile, α-pinene is the only common compound that was detected in all the tested essential oils. 

The significant antimicrobial effect could be attributed to the presence of high percentage of oxygenated compounds specially cedrol (40). The results of antimicrobial activities proved in this study are in agreement with previous studies on the antimicrobial activities of essential oils of the plants under investigation (19, 27). Essential oil of the leaves of *Tetraclinis articulata* from Algeria showed antifungal activities against *Fusarium* species (27). Moreover, the essential oil of *Cupressus sempervirens* from Tunisia inhibited the growth of bacteria, fungi and yeast (19). 

## Conclusion

We believe that the present investigation together with previous studies provide support to the antimicrobial properties of the tested essential oils. They could be used as antimicrobial supplement in the developing countries towards the development of new therapeutic agents. Additional *in-vivo* studies and clinical trials would be needed to justify and further evaluate the potential of these oils as antimicrobial agents.
